# Proliferation, Metastasis, and Radiosensitivity of Nasopharyngeal Carcinoma Cells in the Expression and Effect of Kiwifruit Extract through the Regulation of miR-205-5p

**DOI:** 10.1155/2022/6925772

**Published:** 2022-08-11

**Authors:** Qiuqiu Chen, Min Pan, Yusong Lu, Feifei Wei, Chunqiao Chen, Hui Huang

**Affiliations:** Guilin People's Hospital, Guilin 541002, China

## Abstract

**Background:**

Radix Actinidiae extract (RAE) has been shown to inhibit cancer in many studies, but its potential mechanism in nasopharyngeal cancer (NPC) progression remains unclear.

**Methods:**

NPC cells (SUNE1) were treated with different doses of RAE. For transfection, SUNE1 cells were transfected with the microRNA (miR)-205-5p inhibitor (anti-miR-205-5p) or mimic followed by treatment with 200 *μ*g/mL RAE for 24 h. The MTT assay and colony formation assay were used to detect cell proliferation and radiosensitivity. The transwell assay was used to detect cell migration and invasion. The expression of miR-205-5p was detected by quantitative real-time PCR. The protein expression levels of matrix metalloproteinase-2 (MMP2) and matrix metalloproteinase-9 (MMP9) were detected by western blot analysis.

**Results:**

RAE inhibited NPC cell proliferation, migration, and invasion, while it enhanced radiosensitivity (*P* < 0.05). Also, RAE treatment decreased miR-205-5p expression, as well as MMP2 and MMP9 protein levels (*P* < 0.05). Anti-miR-205-5p transfection enhanced the effects of RAE on NPC cell proliferation, migration, invasion, and radiosensitivity (*P* < 0.05), while miR-205-5p mimic transfection had an opposite effect (*P* < 0.05).

**Conclusion:**

RAE might decrease miR-205-5p, thereby it inhibited NPC cell proliferation and metastasis and enhanced radiosensitivity.

## 1. Introduction

Nasopharyngeal cancer (NPC) is a common malignant tumor in China, which refers to tumor lesions of the nose and throat, and its incidence is increasing year by year [1, 2]. Radiotherapy is the main treatment for NPC, but some patients are prone to developing radiotherapy resistance and thus reducing the therapeutic effect [3, 4]. Distant metastasis is one of the important causes of poor prognosis and death for NPC patients [5, 6]. Therefore, it is of great significance to find drugs that inhibit NPC cell metastasis and enhance radiotherapy sensitivity.

Radix Actinidiae, also known as the root of Actinidia chinensis Planch., is a kind of traditional Chinese medicine with high medicinal value [7]. Studies have shown that Radix Actinidiae extract (RAE) can play an anticancer role by regulating microRNA (miRNA) expression. Previous research had suggested that the RAE could inhibit the proliferation of esophageal cancer cells by upregulating miR-451 expression [8]. However, the effect of RAE on the growth and development of NPC is unknown. MiR-205-5p was upregulated in cisplatin-resistant NPC cells, and its knockdown could inhibit cell epithelial-mesenchymal transition [9]. Besides, upregulated miR-205-5p had been shown to enhance NPC cell angiogenesis and metastasis [10]. In this study, we found that RAE could significantly inhibit miR-205-5p expression, but whether RAE mediates NPC metastasis and radioresistance through regulating miR-205-5p expression remains unclear. Therefore, the present study mainly investigated whether RAE regulated miR-205-5p to participate in regulating NPC progression and radiosensitivity.

## 2. Materials and Methods

### 2.1. Preparation of RAE

RAE (Hengyi Traditional Chinese Medicine Technology Co., LTD., Bozhou, China) was ground into powder, dried, and soaked in n-butanol for 12 h for reflux extraction. The filtrate was collected and then soaked in n-butanol 2 L for reflux extraction. After combining the filtrate twice, n-butanol was recovered by vacuum pressure and drying. Then, RAE was added with distilled water for rotational heating and dissolution to prepare saturated aqueous solution. The concentration of the original solution was 1 g/mL, and the culture solution was added and diluted to 50 *μ*g/mL, 100 *μ*g/mL, and 200 *μ*g/mL.

### 2.2. Experimental Grouping

SUNE1 cells (2 × 10^5^cells/mL, SenBeiJia, Nanjing, China) were seeded into 6-well plates (100 *μ*L/well), then treated with different concentrations (50 *μ*g/mL, 100 *μ*g/mL, and 200 *μ*g/mL) of RAE for 24 h, and named as the 50 *μ*g/mL RAE group, 100 *μ*g/mL RAE group, and 200 *μ*g/mL RAE group. In addition, nontreated SUNE1 cells were used as the normal control (NC) group. Lipofectamine™ 3000 transfection reagent (Invitrogen, Carlsbad, CA, USA) was used to transfect miR-205-5p mimic, the miR-205-5p inhibitor (anti-miR-205-5p), and their negative controls (miR-NC and anti-miR-NC) (synthesized by RiboBio, Guangzhou, China) into SUNE1 cells according to manufacturer's instructions. After transfection, SUNE1 cells were treated with 200 *μ*g/mL RAE for 24 h and denoted as the 200 *μ*g/mL RAE + anti-miR-NC group, 200 *μ*g/mL RAE + anti-miR-205-5p group, 200 *μ*g/mL RAE + miR-NC group, and 200 *μ*g/mL RAE + miR-205-5p group.

### 2.3. MTT Assay

SUNE1 cells in each group were collected and inoculated into a 96-well plate (3 × 10^3^ cells/well), and 20 *μ*L MTT solution (Beyotime, Shanghai, China) was added to each well. The cells were cultured for 4 h, and then the supernatant was discarded. 150 *μ*L DMSO was added to each well, and the absorbance of each well was detected by a microplate reader at 490 nm (A490).

### 2.4. Colony Formation Assay

For measuring colony formation ability, SUNE1 cells were inoculated into a 6-well plate (500 cells/well) and incubated at 37°C in a 5% CO_2_ incubator for 14 days. The culture medium was changed every 3 days. The colonies were fixed by 4% paraformaldehyde (15 min) and stained by crystal violet (5 min). After washing with PBS, the colony numbers were counted.

For assessing radiosensitivity, SUNE1 cells were irradiated with different doses of 0, 2, 4, 6, and 8 Gy of vertical X-ray irradiation. The cells were collected and inoculated in 24-well plates (1 × 10^4^ cells/well). The cells were cultured in a 37°C incubator for 10–15 d, fixed in methanol for 20 min, and stained with crystal violet for 20 min. The number of cell clones and cell survival fraction were calculated, and the sensitization enhancement ratio (SER) was calculated by referring to the click multitarget model.

### 2.5. Transwell Assay

Foe measuring cell migration, SUNE1 cells (1 × 10^5^ cells/mL) were added to the upper chamber (Corning Inc. Corning, NY, USA) at the rate of 200 *μ*L per well, and 600 *μ*L medium containing 10% FBS was added to the lower chamber. The cells were cultured in a 37°C incubator for 24 h, fixed with paraformaldehyde for 20 min, and stained with 0.1% crystal violet solution for 20 min. The number of migrated cells was observed under a microscope. For detecting cell invasion, the Transwell chamber was coated with Matrigel (BD Biosciences, San Jose, CA, USA) and fixed in a 37°C incubator for 5 h. Subsequent experiment procedure was the same as the migration experiment.

### 2.6. Quantitative Real-Time PCR (qRT-PCR)

Total RNA was extracted from each group of SUNE1 cells with the RNA extraction reagent (TransGen Biotech, Beijing, China). The concentration and purity of RNA were detected by using a Nanodrop2000 c ultra-microspectrophotometer, and then RNA was synthesized into cDNA by the cDNA First Strand Synthesis Kit (Thermo Fisher Scientific, Waltham, MA, USA). PCR amplification reaction system: SYBR Green Master Mix (Thermo Fisher Scientific) 10 *μ*L, forward and reverse primer 0.8 *μ*L, cDNA 2 *μ*L, ddH_2_O complement system to 20 *μ*L. Reaction conditions: 95°C for 2 min, 95°C for 15 s, 60°C for 1 min, and 72°C for 30 s (cycle 40 times). The relative expression of miR-205-5p was calculated by the 2^−ΔΔCt^ method with U6 as an internal reference.

### 2.7. Western Blot

SUNE1 cells were added with RIPA lysis buffer (Beyotime) to extract total protein. Protein concentration was determined by the BCA method (Beyotime). Protein was isolated by SDS-PAGE gel, transferred PVDF membrane, and sealed with 5% skim milk for 2 h. The membrane was hatched with anti-MMP2 (1 : 800, Abcam, Cambridge, MA, USA), anti-MMP9 (1 : 800, Abcam), and internal reference anti-*β*-actin (1 : 1000, Abcam) for 24 h and then hatched with the secondary antibody (1:3000, Abcam) for 1 h. The darkroom exposure was developed using the ECL reagent (Beyotime), and the gray values of each band were analyzed by ImageJ software with *β*-actin as loading control.

### 2.8. Statistical Analysis

SPSS21.0 statistical software was used to analyze the data, and the data were expressed as *x* ± *s* and were in line with normal distribution. An independent sample *t*-test was used for comparison between the two groups, and one-way ANOVA was used for comparison between multiple groups. *P* < 0.05 was considered statistically significant.

## 3. Results

### 3.1. RAE Inhibited SUNE1 Cell Proliferation

**Figure 1:**
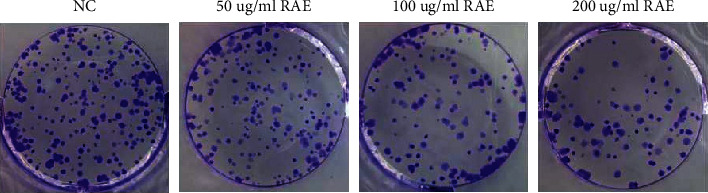
Effects of different concentrations of RAE on the colony number of SUNE1 cells.

Compared to the NC group, cell viability and colony numbers were markedly reduced in the 50 *μ*g/mL RAE group, 100 *μ*g/mL RAE group, and 200 *μ*g/mL RAE group (*P* < 0.05) (Figure 1 and Table 1). These data confirmed that RAE could suppress NPC cell proliferation.

### 3.2. RAE Reduced SUNE1 Cell Migration and Invasion

**Figure 2:**
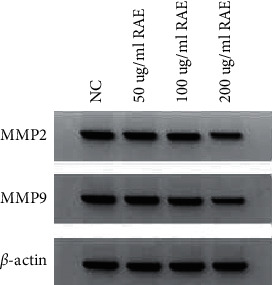
Effects of different concentrations of RAE on the MMP2 and MMP9 protein expression of SUNE1 cells.

Compared to the NC group, the migrated cell numbers, invaded cell numbers, and the protein expression of MMP2 and MMP9 were significantly reduced in the 50 *μ*g/mL RAE group, 100 *μ*g/mL RAE group, and 200 *μ*g/mL RAE group (*P* < 0.05) (Figure 2 and Table 2). These data suggested that RAE inhibited NPC cell metastasis.

### 3.3. RAE Enhanced the Radiosensitivity of SUNE1 Cells

**Figure 3:**
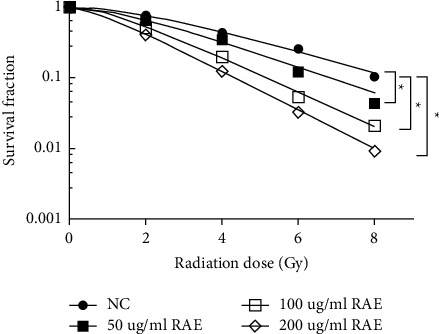
Effects of different concentrations of RAE on SUNE1 cell survival fraction.

Compared to the NC group, cell survival fraction was remarkably inhibited (*P* < 0.05), and SER was markedly increased (*P* < 0.05) in the 50 *μ*g/mL RAE group, 100 *μ*g/mL RAE group, and 200 *μ*g/mL RAE group (Figure 3 and Table 3). All data indicated that RAE could promote the radiosensitivity of NPC cells.

### 3.4. RAE Decreased miR-205-5p Expression in SUNE1 Cells

Compared to the NC group, miR-205-5p expression was significantly decreased in the 50 *μ*g/mL RAE group, 100 *μ*g/mL RAE group, and 200 *μ*g/mL RAE group (Table 4).

### 3.5. Anti-miR-205-5p Aggravated the Effect of RAE on SUNE1 Cell Proliferation, Metastasis and Redioresistance

**Figure 4:**
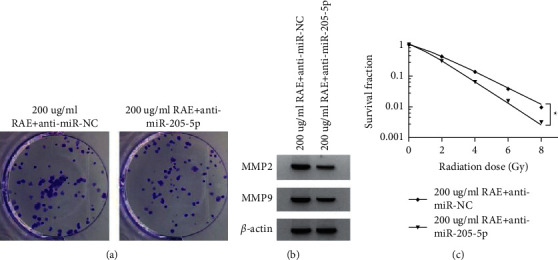
Effects of anti-miR-205-5p on RAE-induced SUNE1 cell functions. (a) The colony formation assay measured colony numbers; (b) WB analysis detected the expression of MMP2 and MMP9; (c) the colony formation assay detected cell survival fraction.

Compared to the 200 *μ*g/mL RAE + anti-miR-NC group, cell viability, colony numbers, the migrated cell numbers, the migrated cell numbers, the protein expression of MMP2 and MMP9, and cell survival fraction were significantly reduced (*P* < 0.05) in 200 *μ*g/mL RAE + anti-miR-205-5p, and SER was 1.331 (Figures 4(a)–4(c) and Tables 5-6).

### 3.6. MiR-205-5p Suppressed the Effect of RAE on SUNE1 Cell Proliferation, Metastasis, and Redioresistance

**Figure 5:**
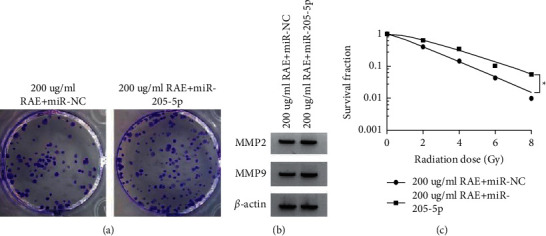
Effects of miR-205-5p on RAE-induced SUNE1 cell functions. (a) The colony formation assay measured colony numbers; (b) WB analysis detected the expression of MMP2 and MMP9; (c) the colony formation assay detected cell survival fraction.

Compared to the 200 *μ*g/mL RAE + miR-NC group, cell viability, colony numbers, the migrated cell numbers, the migrated cell numbers, the protein expression of MMP2 and MMP9, and cell survival fraction were significantly enhanced (*P* < 0.05) in 200 *μ*g/mL RAE + anti-miR-205-5p, and SER was 1.331 (Figures 5(a)–5(c) and Tables 7-8).

## 4. Discussion

Molecular targeted therapy plays an important role in many cancers [11, 12]. Therefore, exploring the biological behavior of a tumor from the molecular level and finding new therapeutic methods can achieve the purpose of inhibiting tumor growth. Studies have shown that some Chinese medicines have the effect of antitumor formation of NPC and can inhibit NPC development through a variety of ways [13, 14]. miRNA plays an important regulatory role in the development and progression of NPC and may serve as a potential therapeutic target for NPC [15, 16]. However, whether miRNA can be used as a potential target of traditional Chinese medicine against NPC remains to be further explored.

Previous studies had shown that RAE repressed breast cancer cell proliferation and induced apoptosis by inhibiting the activation of the VEGF/PI3K/AKT signaling pathway [17]. RAE had been shown to inhibit the proliferation, migration, and invasion of hepatocellular carcinoma cells by upregulating miR-34-5p expression [18]. Besides, RAE could inhibit proliferation and promote apoptosis of cervical cancer cells [19]. Our study showed that RAE could reduce the viability and the colony numbers of NPC cells, suggesting that RAE hindered NPC cell proliferation. MMP2 and MMP9 are matrix metalloproteinases, whose upregulated expression can promote cell metastasis by regulating extracellular matrix deposition [20]. The results of this study showed that the migrated and invaded cell numbers and the protein levels of MMP2 and MMP9 were decreased after RAE treatment, suggesting that RAE could inhibit NPC cell migration and invasion. Meanwhile, the survival fraction of NPC cells were decreased, and the SER ratio was increased after RAE treatment, confirming that RAE enhanced NPC cell radiosensitivity.

MiR-205-5p functioned as a tumor promoter in many cancers, including ovarian cancer [21] and lung cancer [22]. On the contrary, many studies showed that miR-205-5p was downregulated in thyroid cancer, and its upregulation suppressed cell proliferation and invasion [23]. In an NPC-related study, miR-205-5p had been found to be a tumor promoter [9, 10]. Here, we found that miR-205-5p expression in NPC cells was decreased after RAE treatment, suggesting that RAE might play an anti-NPC role by inhibiting miR-205-5p expression. Moreover, downregulated miR-205-5p could enhance the effect of RAE on NPC cell proliferation, metastasis, and radiosensitivity, while upregulation of miR-205-5p could reduce the effect of RAE on NPC cell functions, confirming that RAE could inhibit NPC cell proliferation and metastasis and enhance radiosensitivity by regulating miR-205-5p.

## Figures and Tables

**Table 1:** Effects of different concentrations of RAE on the viability and colony number of SUNE1 cells (*n* = 9).

Groups	A490	Colony numbers
NC	1.042 ± 0.09	118 ± 8.25
50 *μ*g/mL RAE	0.837 ± 0.08^*∗*^	90 ± 5.16^*∗*^
100 *μ*g/mL RAE	0.514 ± 0.04^*∗*^	65 ± 3.17^*∗*^
200 *μ*g/mL RAE	0.435 ± 0.03^*∗*^	46 ± 3.02^*∗*^

*Note.* Compared to the NC group, ^*∗*^*P* < 0.05.

**Table 2:** Effects of different concentrations of RAE on SUNE1 cell migration and invasion (*n* = 9).

Groups	Migrated cell numbers	Invaded cell numbers	MMP2	MMP9
NC	237 ± 18.43	154 ± 11.25	0.84 ± 0.07	0.73 ± 0.06
50 *μ*g/mL RAE	196 ± 15.03^*∗*^	121 ± 9.02^*∗*^	0.68 ± 0.05^*∗*^	0.62 ± 0.05^*∗*^
100 *μ*g/mL RAE	164 ± 13.07^*∗*^	79 ± 6.18^*∗*^	0.48 ± 0.03^*∗*^	0.53 ± 0.04^*∗*^
200 *μ*g/mL RAE	108 ± 8.36^*∗*^	61 ± 4.15^*∗*^	0.37 ± 0.02^*∗*^	0.39 ± 0.02^*∗*^

*Note.* Compared to the NC group, ^*∗*^*P* < 0.05.

**Table 3:** Parameter values of the single-click multitarget model of RAE treatment combined with X-ray irradiation on SUNE1 cells (*n* = 9).

Groups	*D*_0_ (Gy)	*D*_*q*_ (Gy)	*N*	SF2	*k*	SER (do ratio)
NC	2.837	2.072	2.076	0.757	0.353	—
50 *μ*g/mL RAE	2.333	1.532	1.928	0.655	0.429	0.822
100 *μ*g/mL RAE	1.765	1.149	1.917	0.525	0.567	—
200 *μ*g/mL RAE	1.583	0.712	1.568	0.406	0.632	1.115

*Note.* Compared to the NC group, ^*∗*^*P* < 0.05.

**Table 4:** Effects of different concentrations of RAE on miR-205-5p expression in SUNE1 cells (*n* = 9).

Groups	miR-205-5p
NC	1.00 ± 0.12
50 *μ*g/mL RAE	0.81 ± 0.08^*∗*^
100 *μ*g/mL RAE	0.63 ± 0.05^*∗*^
200 *μ*g/mL RAE	0.45 ± 0.03^*∗*^

*Note.* Compared to the NC group, ^*∗*^*P* < 0.05.

**Table 5:** Anti-miR-205-5p enhanced the suppressive effect of RAE on SUNE1 cell proliferation, migration and invasion (*n* = 9).

Groups	miR-205-5p	A490	Colony number	Migrated cell numbers	Invaded cell numbers	MMP2	MMP9
200 *μ*g/mL RAE + anti-miR-NC	1.00 ± 0.08	0.439 ± 0.03	49 ± 3.27	110 ± 8.13	65 ± 5.25	0.35 ± 0.03	0.38 ± 0.03
200 *μ*g/mL RAE + anti-miR-205-5p	0.38 ± 0.03^*∗*^	0.153 ± 0.02^*∗*^	28 ± 1.86^*∗*^	73 ± 5.43^*∗*^	38 ± 2.54^*∗*^	0.15 ± 0.02^*∗*^	0.12 ± 0.02^*∗*^

*Note.* Compared to the 200 *μ*g/mL RAE + anti-miR-NC group, ^*∗*^*P* < 0.05.

**Table 6:** Parameter values of the single-click multitarget model of RAE treatment and transfection combined with X-ray irradiation on SUNE1 cells.

Groups	*D*_0_ (Gy)	*D*_*q*_ (Gy)	*N*	SF2	*k*	SER (do ratio)
200 *μ*g/mL RAE + anti-miR-NC	1.653	0.692	1.520	0.416	0.605	—
200 *μ*g/mL RAE + anti-miR-205-5p	1.241	0.594	1.614	0.302	0.806	1.331

*Note.* Compared to the 200 *μ*g/mL RAE + anti-miR-NC group, ^*∗*^*P* < 0.05.

**Table 7:** miR-205-5p reduced the effect of RAE on SUNE1 cell proliferation, migration, and invasion (*n* = 9).

Groups	miR-205-5p	A490	Colony numbers	Migrated cell numbers	Invaded cell numbers	MMP2	MMP9
200 *μ*g/mL RAE + miR-NC	0.95 ± 0.10	0.434 ± 0.04	47 ± 3.58	105 ± 9.03	64 ± 4.63	0.34 ± 0.02	0.40 ± 0.03
200 *μ*g/mL RAE + miR-205-5p	0.80 ± 0.08^*∗*^	0.866 ± 0.07^*∗*^	98 ± 6.38^*∗*^	196 ± 15.36^*∗*^	139 ± 10.87^*∗*^	0.78 ± 0.08^*∗*^	0.75 ± 0.06^*∗*^

*Note.* Compared to the 200 *μ*g/mL RAE + miR-NC group, ^*∗*^*P* < 0.05.

**Table 8:** Parameter values of the single-click multitarget model of RAE treatment and transfection combined with X-ray irradiation on SUNE1 cells.

Groups	*D*_0_ (Gy)	*D*_*q*_ (Gy)	*N*	SF2	*k*	SER (do ratio)
200 *μ*g/mL RAE + miR-NC	1.806	0.493	1.314	0.410	0.554	—
200 *μ*g/mL RAE + miR-205-5p	2.280	1.559	1.981	0.655	0.439	0.792

*Note.* Compared to the 200 *μ*g/mL RAE + miR-NC group, ^*∗*^*P* < 0.05.

## Data Availability

The labeled dataset used to support the findings of this study is available from the corresponding author upon request.
